# How mathematical epidemiology became a field of biology: a commentary on Anderson and May (1981) ‘The population dynamics of microparasites and their invertebrate hosts’

**DOI:** 10.1098/rstb.2014.0307

**Published:** 2015-04-19

**Authors:** J. A. P. Heesterbeek, M. G. Roberts

**Affiliations:** 1 Department of Farm Animal Health, Faculty of Veterinary Medicine, University of Utrecht, Yalelaan 7, Utrecht 3584 CL, The Netherlands; 2 Institute of Natural and Mathematical Sciences, New Zealand Institute for Advanced Study and the Infectious Disease Research Centre, Massey University, Private Bag 102 904, North Shore Mail Centre, Auckland, New Zealand

**Keywords:** mathematical modelling, infectious diseases, epidemiology, population dynamics, ecology, invertebrates

## Abstract

We discuss the context, content and importance of the paper ‘The population dynamics of microparasites and their invertebrate hosts’, by R. M. Anderson and R. M. May, published in the *Philosophical Transactions of the Royal Society* as a stand-alone issue in 1981. We do this from the broader perspective of the study of infectious disease dynamics, rather than the specific perspective of the dynamics of insect pathogens. We argue that their 1981 paper fits seamlessly in the systematic study of infectious disease dynamics that was initiated by the authors in 1978, combining effective use of simple mathematical models, firmly rooted in biology, with observable or empirically measurable ingredients and quantities, and promoting extensive capacity building. This systematic approach, taking ecology and biology rather than applied mathematics as the motivation for advance, proved essential for the maturation of the field, and culminated in their landmark textbook of 1991. This commentary was written to celebrate the 350th anniversary of the journal *Philosophical Transactions of the Royal Society*.

## 1. Introduction and historic context

The value of mathematical models for understanding the dynamics and control of infectious disease was recognized more than two centuries ago. In 1766, Daniel Bernoulli, working on a mathematical analysis of the benefits of smallpox inoculation, wrote ‘in a matter which so closely concerns the well-being of the human race, no decision shall be made without all knowledge which a little analysis and calculation can provide’ [1]. Bernoulli's analysis, read to the French Academy of Sciences in 1760, addressed a topic hotly debated in society and government at the time—the value of ‘variolation/inoculation’. Using the first known model of infectious disease dynamics, he showed that despite the risks to individuals, inoculation with smallpox was beneficial for society as a whole as it increased average life expectancy by more than three years, even discounting the additional deaths the preventive measure would cause. Almost 250 years after Bernoulli's publication, infectious disease dynamics has grown into a field of science, with an established core of approaches and methods and a suite of case law and generic insights gathered by studying specific infectious agents, as well as general epidemiological phenomena in animals, plants and humans. Its history is rich in the sense that many authors have explored a broad range of questions throughout the three centuries.

Although substantial work exists from elsewhere, many important developments of lasting value towards building a body of methods and insights originated in the UK in the nineteenth and early twentieth centuries. In fact, essential groundwork was already started there much earlier at the start of the seventeenth century, when data on morbidity, mortality, cause of death and population size were starting to be routinely collected, at first sparked by outbreaks of plague, and later used, for example, in calculations of life expectancy. Notably John Gaunt in his use of the ‘bills of mortality’ was an important pioneer [2], as was Edmund Halley, who developed the first life table and published it in the *Philosophical Transactions* *of the Royal Society* in 1693 [3]. Bernoulli in 1760 used Halley's life table as the basis in his calculations of the expected change in average life expectancy under inoculation against smallpox. In the mid-nineteenth century, William Farr, Registrar General in the UK, was instrumental in gathering quality data and using these insightfully, for example, in his analysis of ‘the cattle plague’ (rinderpest) [4].

Our aim is not to provide a historic overview, but it is not without foundation to state that the UK played a dominant role in the field's genesis. It therefore makes sense to focus on the developments in the UK to sketch the ‘line of descent’ of the main proponents of our paper, Robert M. May and Roy M. Anderson, and the tradition out of which their contributions arose. A brief historical sketch up to 1975 can be found in the seminal book by Bailey [5], the first substantial textbook to provide an overview of the budding field, and published just before May and Anderson started to work on the topic.

The great majority of work on infectious disease dynamics before the start of the twentieth century was driven by the desire to understand specific infectious diseases and specific public health problems. This tradition culminated in the elaborate work of Ronald Ross, who received the second Nobel Prize in Medicine for his work on the transmission of malaria. Ross wrote that the epidemiology of infectious diseases must be considered mathematically, and that the mathematical method of treatment is really nothing but the application of careful reasoning to the problems at issue [6]. Ross introduced the fundamental insight that not all mosquitoes had to be eliminated to stop the malaria parasite from spreading, but that depression of the number of mosquitoes per human host in a population to a value below a critical level was sufficient. He expressed that critical level using parameters that could be measured in the field, and based on a model describing the proposed mechanisms of the parasite transmission process. Ross's ideas about applying mathematical reasoning to infectious disease dynamics originated from his ambition to understand malaria transmission and control. However, he was the first to develop, in an appendix (called the ‘theory of happenings’) to his 1911 book and in subsequent papers with Hilda Hudson, a general theory (which he called ‘a priori pathometry’) of infectious disease dynamics not specifically tailored to a particular pathogen or public health problem [7,8]. This marks the start of infectious disease dynamics as a scientific field, with its own research philosophy and set of tools, and it is this tradition that Anderson and May would come to systematically explore and expand. Ross's ideas on thresholds and critical community size play a large role in the way of thinking that Anderson and May adopted.

The work by Ross sparked the interest of more theoretically inclined researchers, resulting in decades of progress on mathematical tools and analysis, not specific to particular diseases or public health problems. A series of influential papers by McKendrick and Kermack in the 1930s generalized Ross's initial ideas of critical thresholds for malaria to critical size of a community of susceptible individuals necessary for an infectious disease to become established in a population [9]. Mathematicians and statisticians started to dominate the field, with most contributors from the UK and the USA. The British mathematicians Maurice Bartlett and Norman Bailey had enormous influence in the 1950s, 1960s and 1970s, both contributing to stochastic models for infectious diseases [5,10]. Roy Anderson was a postdoctoral fellow with Bartlett at the Department of Biomathematics in Oxford in the early 1970s, possibly the first department of its kind. Bartlett established the concept of a critical community size for outbreaks to occur [11]. This plays a large role in the analysis and discussion of results in the subject of our review, the paper ‘The population dynamics of microparasites and their invertebrate hosts’, by R. M. Anderson and R. M. May, published in the *Philosophical Transactions of the Royal Society* as a stand-alone issue in 1981 [12].

A concept related to that of the critical community size that has played a pivotal role in mathematical epidemiology is the basic reproduction number, 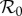, called the ‘basic reproductive rate’ in the Anderson & May paper and other papers from that period, and denoted there by *R*. It is defined as the average number of new cases of an infection caused by a single infected individual in a population consisting only of susceptible individuals, mirroring similar concepts in demography [13,14]. The idea of 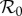 arose from the work by Ross on the critical threshold for malaria, but was implicit there. It was developed into a well-defined epidemiological quantity mainly by the zoologist George Macdonald (working at the London School of Hygiene and Tropical Medicine), again in the context of malaria [15]. When 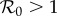 an infectious agent entering a population of susceptible individuals will cause an epidemic; when 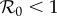 the infectious agent cannot spread in that population. The concept of the basic reproduction number is both simple and powerful and has become one of the most used and useful ideas in understanding infection dynamics. Under certain assumptions on how transmission opportunities in a population (i.e. the number of contacts where infection could be transmitted) change with increasing population size, one can interpret the condition 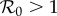 as being equivalent to the condition that the size of the susceptible population into which the infectious agent is introduced is larger than the critical community size. These properties are exploited by Anderson and May throughout their paper to explain the results of their models. The concept of 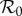 is closely linked to quantities such as ‘net fertility’ or ‘net reproductive rate’ in demography (introduced mainly through the work of Alfred Lotka), and ‘absolute fitness’ or ‘reproductive fitness’ in population genetics (introduced mainly through the work of Ronald Fisher and Sewall Wright), although these concepts did not evolve from each other in a linear manner [14]. They all describe the average contributions of members of a given generation to the next generation, in terms of new infections caused, the birth of daughters, or genotypes produced.

The advances that Anderson and May brought to the growing field were twofold. First, their main aim was to explain epidemiological patterns that could be observed without concentrating on any one infectious agent in particular. This differs from the generic approach taken in the decades before, because the patterns they sought to understand were taken from empirical observations and data; and the models they developed were firmly rooted in biological assumptions about mechanisms that could be behind the observed patterns. Possibly, this deviation in approach can be understood from their primary interest that did not come from mathematics or medicine, but from zoology and ecology.

The second advance was that, in contrast to the situation so far where researchers worked mainly in isolation, Anderson and May collaborated in larger groups with biologists and mathematicians, thereby establishing and educating the first real generation of dedicated epidemiological modellers. Many of their earlier collaborators and students are influential to the present day, having gained professorships and contributed further generations of students and colleagues. One can certainly speak of a dynasty of researchers in infectious disease dynamics, started by Anderson and May and now scattered over several continents. From its beginnings in understanding public health problems, Anderson and May brought the maturing field back from the once essential direction of mathematical abstraction, and firmly steered it into being a field of biology rather than applied mathematics. The driving forces became the understanding of empirically observed biological patterns, and the need for evidence-based public health decisions, for which a mathematical approach became essential.

To appreciate their approach and philosophy, it is historically important to emphasize the context in which their collaboration evolved and their careers developed, as well as the role May played in the ‘golden age’ of theoretical ecology. Both May and Anderson were part of a very active group of ecologists, entomologists, zoologists and theoreticians that arose under the guidance of Richard Southwood at the Silwood Park campus of Imperial College London. The activities and influence of this group has recently been documented [16], allowing us to place the Anderson & May paper in perspective.

By the time they met in the summer of 1973, May had just been appointed to a chair at Princeton University and had completed his seminal book *Stability and Complexity in Model Ecosystems* [17]. This and subsequent work opened up an entirely new way of looking at phenomena in the natural world in terms of nonlinear dynamic behaviour that could be generated from simple models [18]. Silwood Park was very attractive to May, and he established close and diverse collaborations there. There were field and empirical ecologists, entomologists and zoologists, with interesting data and problems, and who were very welcoming to a theoretician who wanted to firmly root theory in real ecology [16,19]. Anderson, a zoologist working on helminth parasites of fish, had started using mathematical models linked to empirical data and observations. Epidemiological data, particularly from parasite and invertebrate systems, were available and this possibly made the combination of epidemiology and ecology an attractive one for both May and Anderson. It seems only natural that they would strike up a close collaboration, which would turn out to be the most fruitful one of their long (and continuing) careers. Their paper in *Philosophical Transactions* [12] is the fifth in, what in hindsight can be called, a series of careful, systematic, almost tutorial-like papers that established the authors' names and influence early in their prolific collaboration, clearly outlining a structured philosophy in research into infectious disease dynamics, and culminating in the comprehensive textbook on infectious disease modelling in 1991 [13].

The first papers in this series were two connected publications in the *Journal of Animal Ecology* [20,21] dealing with macroparasites (broadly speaking worm parasites or helminths) of vertebrate hosts, together comprising 50 printed pages, and addressing the possibilities for regulation of the host population dynamics by the parasite. This seems a natural starting point for the collaboration, given Anderson's origins and interests in studying helminths, and May's interest in the stability of ecological systems. These were followed a year later by a new pair of connected publications, this time in *Nature*, reviewing the state of the art of the dynamics of both macroparasites and microparasites (broadly speaking, viruses, bacteria, protozoa) of vertebrate hosts [22,23].

In all of these papers, their approach originated from an ecological interest, rather than an epidemiological interest. The point is that both for ecology (where parasites and pathogens were largely ignored at the time and the emphasis was on understanding predator–prey interactions), and for (mathematical) epidemiology (where progress in understanding was guided by either interesting mathematical problems generated by unspecified infectious agents, or by assumptions that were relevant to the human context), their way of thinking brought entirely new perspectives. Assumptions such as a constant host population size were no longer tenable when looking at infections in populations other than human. Relaxing this assumption was not only a natural step when coming at this topic from ecology rather than medicine, it also opened the way to asking new questions about infectious disease dynamics and studying a much richer set of observed phenomena. Once taken, these steps also turned out to be essential for understanding infectious disease dynamics in human populations.

The step to extend the initial sets of papers to address the regulation of invertebrates by microparasites is a natural one, as May had already worked on parasitoids of insects with Michael Hassell at Silwood Park [24], where there was a clear interest in the topic. An illustration of this interest is that the CABI Institute of Biological Control moved to Silwood Park in 1981 [16]. Also from the point of view of empirical data with interesting unexplained phenomena and patterns, the invertebrates and their infectious agents must have appealed as a promising topic. For example, the observation that forest insect pests, such as the larch budmoth, show long-period cycles (8–9 years) of population explosion, had already fascinated ecologists for a number of years, and there were several competing hypotheses [25]. Anderson and May added a hypothesis of their own, leading to extensive and heated debate among ecologists in the years following its publication. Another issue that was gaining prominence at the time was the potential use of infectious agents as a means of biological control of invertebrate pest species. These ecological and applied issues can only be meaningfully investigated with models that allow for a varying population size. In the next section, we give a brief overview of the paper.

## 2. The population dynamics of microparasites and their invertebrate hosts

The style and content of the paper [12] fit naturally in the approach established by Anderson and May in their previous sets of matching papers ([20,21] and [22,23]) but this was their longest and most structured publication so far. It is 74 pages in print, including technical appendices and references, and the editors of *Philosophical Transactions* are to be commended on accepting the paper despite its non-standard length. Robert May in his cover letter (undated, but of 18 April 1980 according to [12]) expressed the hope that it ‘might be published as a free-standing issue’, but there was of course no guarantee. The alternative would have been to split the paper, as they had done previously. However, in contrast to the two earlier submissions, there is not a natural point in the paper to make the cut.

As an aside, it is interesting to note that although this paper is a very recent one in this anniversary collection of the *Philosophical Transactions*, a lot has changed in the 35 years since. For example, a substantial part of the paper would now be published as electronic supplementary material. In producing the paper and its results, calculations were done on a hand-held calculator programmable with a magnetic strip reader, and graphs were drawn by hand and turned into figures with Letraset (R. M. Anderson 2014, personal communication). For the journal, finding suitable reviewers was a problem. Those suggested by the authors were both from the Silwood Park group, and perhaps not seen as sufficiently independent. In any case, according to the journal archive they were not approached. Other names suggested by the editor, among them Bartlett and Kendall, were unavailable. The single (!) reviewer who eventually assessed the manuscript has passed away, and can be revealed to be John Maynard Smith. The referee report (dated 20 June 1980) reads (in full): ‘Professor Harper asked me if I would look at the enclosed manuscript. I think it is entirely suitable for publication in the ‘Transactions’. It is an important contribution to knowledge. It is clearly written, and as brief as it could be in the light of the field covered’. Although substantial discussion about assumptions, choices and conclusions would have been warranted, it is possibly only with today's knowledge that any profound criticism can be formulated. Maynard Smith was by no means an expert in infectious disease modelling, but when the paper was submitted there was relatively little modelling in infectious diseases that went beyond simple, low-dimensional models of homogeneous populations.

The paper is didactically well thought out, carefully written and comprehensive. It presents a study and systematic exploration of the influence of infectious agents on populations of invertebrate hosts that are varying in size, carefully exploiting the possibilities of simple models. It provides a set of tools and an approach that set the stage for a vast amount of subsequent work. It is a tutorial, explaining biological assumptions, observed phenomena to be studied, terminology and mathematics in detail. Its way of thinking goes far beyond speaking only to invertebrate species and their pathogens but is exemplified by the urge to explain and understand observed phenomena and patterns in biological terms, using models as tools. Careful use of low-dimensional models, staying close to data and biology, using analytical results for robustness, clever use of figures to highlight changes in the stability of steady states in parameter space, translating these results into biological terms and (in hindsight) intuition for increased understanding are all hallmarks of its philosophy.

The authors’ approach was influential, not necessarily because they were always right, but because biologists could relate to the careful reasoning. The models were explained so that they could be understood by biologists, they had links to data, and despite their simplicity generated relevant and often surprising insight. Also, concepts and new ways of looking at things were introduced and/or brought to prominence. The authors may not have always been the first, but many ideas and techniques only gained traction once they showed how they could be used effectively.

### (a) The fundamental philosophy of the approach

The first 10 pages of the paper are spent carefully setting the scene for modelling the interaction between parasites and their invertebrate hosts. The model discussed would now be referred to as *SIS* with constant population size, in other words a logistic differential equation. The model is justified by comparison with data from an experimental epidemic that follows the familiar sigmoid curve over time. The authors recast the model in terms of prevalence of infection (proportion infected), *y*(*t*′), for a rescaled time variable, and obtain
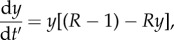
where the dimensionless parameter
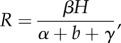
with *H* the (constant) population size, *b* the mortality rate of uninfected hosts, *α* + *b* the mortality rate of infected hosts, and *γ* the rate at which infected hosts recover and become susceptible. A selection of parameter values for *b* and *α* is tabulated, it is pointed out that the *transmission parameter *β* is the most difficult to measure, and that *β*H* has units 1/time.

Two cases are then distinguished (excluding *R* = 1):

if *R* > 1 the infection persists within the host population, and the prevalence approaches the value *y* = 1 − 1/*R* over time; and
if *R* < 1 the infection cannot persist, and the prevalence approaches zero.

The authors observe that

*R* is defined to be the expected number of secondary infections (*βH*) produced within the infectious period, 1/(*α* + *b* + *γ*), of one newly introduced host. That is, *R* is the basic reproductive rate of the parasite … , precisely analogous to the conventional ecologists' and demographers' ‘expected number of offspring’, *R*_0_…. (p. 460)

This is one of the earliest references in the work of Anderson and May to what is now the accepted concept, the basic reproduction number (or ratio), 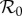, discussed in our introduction. At the time, its use was increasing in the analysis of simple epidemiological models, for example through the work of Klaus Dietz [26]. Somewhat surprisingly, the concept does not feature at all in Anderson and May's influential set of *Nature* papers [22,23].

The authors define the threshold host density, *H*_T_, as that value of *H* for which *R* = 1. Clearly, *H* > *H*_T_ implies *R* > 1 and the infection persists, so *H*_T_ may be interpreted as the critical community size. Although the authors are never explicit about whether *H* is the number of hosts in the population or a population density, they refer to a closed population and are careful to be consistent with units. The logistic model is useful for illustrating the concepts of *R* and *H*_T_, but has the curious property that deaths due to infection are compensated by increased birth rates, to keep the population constant. For the rest of their paper, the authors ‘break new ground’ by treating *H* as a dynamic variable. The idea is introduced in their discussion of ‘Model A’, the most basic of the seven models the authors discuss: Models A–G.

If host population size is not constant, one can meaningfully address the question of *regulation*. In the present context, this means the question whether the infectious agent is able to markedly influence the population dynamics of the host. For example, for host populations that would grow exponentially in the absence of the infectious agent, it may be that they continue to grow but at a slower rate, that they stop growing and reach an equilibrium level, or that they exhibit more complicated behaviour such as oscillations in size.

### (b) Basic dynamics of host–parasite associations: Model A

In their ground-breaking paper from 1978 [20], the authors demonstrated that parasites could regulate a wild animal population. Starting with Model A, they investigate a similar concept in their 1981 paper—could a pathogen regulate an insect population? Assuming that the host population would grow exponentially at rate *r* in the absence of infection, with infection increasing the host mortality rate by *α* as before, they show that if *α* > *r* then the pathogen would regulate the host population size at a level
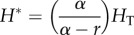
with prevalence of infection y* = *r*/*α*. They also show that if *α* < *r*, then the host population would continue to grow, becoming exponential with rate *r* − *α*, with the prevalence of infection tending to one. The authors remark that the ‘run away’ behaviour of the host population is a consequence of the omission of other density-dependent constraints, such as resource limitation, from the model.

In illustrating the dependence of H* and y* on model parameters (Fig. 6 in [12]), the authors note that if a pathogen were to be selected for biological control, rather than seeking the most pathogenic (highest value of *α*), an intermediate value would result in the lowest population density. This optimum value 
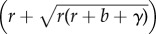
 is not given explicitly. In their discussion of Fig. 6, they remark that a large *β* is required for persistence when host populations turn over rapidly. This statement refers to the definition of *H*_T_, which is inversely proportional to *β*. Reference to Fig. 6 shows that parasite abundance, H*y*, is also inversely proportional to *β* when other parameters are fixed.

In this section, the authors' main concern is whether ‘natural populations of invertebrates typically have microparasitic infections capable of regulating them’ (p. 466). They acknowledge that few field studies have provided estimates of *α* and *r*, and although they present results from laboratory studies showing sufficiently large *α* (table 1 in [12]), they concede that these results may not apply to natural populations. So, although the model shows regulation to be feasible, and the data are encouraging, the cautious conclusion is that infections may ‘contribute, wholly or in part, to the regulation of their invertebrate host populations’ (p. 467).

### (c) Elaborations on the model: Models B–F

The next five sections of the paper are devoted to generalizations of Model A, denoted by Models B–F. The added complications introduced in these sections do not, in the main, greatly change the conclusions from Model A. Models B and C include parasite-induced reduction of host reproduction, and vertical transmission of the pathogen, respectively. Of more interest is Model D, which includes a latent period of infection. Certain combinations of parameter values are shown to lead to stable periodic solutions instead of equilibrium values. However, in the absence of any available data to suggest this occurs for natural host–pathogen systems, the discussion is treated as of mathematical interest and confined to an appendix. For Model E, host stress due to overcrowding is linked with increased pathogenicity. This is modelled by replacing *α* in Model A with 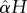. The authors conclude that ‘when pathogenicity is related to host abundance, the parasite will always be capable of regulating population growth, and its main problem is to transmit itself fast enough to counter-balance the rapid death of infected hosts’ (p. 474).

In Model F, the authors introduce a second density-dependent constraint independent of the pathogen, by replacing the host mortality rate *b* with *b*_0_ + *sH*. Hence, in the absence of the pathogen the host population dynamics are governed by a logistic equation, and *H* tends to the carrying capacity *K*. Analysis of the model then leads the authors to conclude that the disease can be maintained if the ‘basic reproductive rate’ *R* is greater than one, where
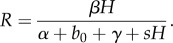


They conclude that this requires *β* > *s* and *H*_T_ < *K*, where
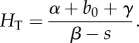


It is argued that if *H* > *K* then the logistic dynamics will reduce *H* below *K*, but *H* < *H*_T_ implies *R* < 1. What is overlooked in this analysis is that *H* is a time-dependent state variable, not a constant. If we define the basic reproduction number to be
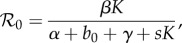
then 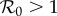 implies both *β* > s and *H*_T_ < *K*, hence the pathogen can persist in the population.

Fig. 13 of the paper [12] shows a (*β*, *K*) section through parameter space, and the locus of 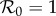 defining the boundary between parasite extinction and persistence. A new concept is defined: *d* = 1−H*/*K* measures the degree to which the host population size is depressed below the disease-free level by the infection. It is shown that maximum *d*, and hence optimal sustainable control of the invertebrate population, is achieved with an intermediate value of pathogenicity *α*.

### (d) Free-living infective stages: Model G

In presenting Model G the authors return to the initial format of Model A, and then modify it to include a free-living stage. This is achieved by adding an equation for the ‘population of free-living infective stages’, *W*, assuming that an infected individual produces these stages at a rate *λ*, and a susceptible individual becomes infected at rate *vW* instead of the mass-action rate *βY*. In the exposition, the units of *W* are not specified. However, the requirement that during the infection process the free-living stages are removed at a rate *vH* implicitly determines the units of *λ* and hence *W*. This observation calls into question the authors' claim based on data in their Table 5 that *λ* is ‘always vastly greater than *α* + *b* + *γ*’ (p. 481), but the assumption simplifies the exposition without materially affecting the results. The authors provide a comprehensive analysis as an appendix. The major result from this section is presented in their Fig. 16. Four (*α*, *λ*) sections through parameter space each show four regimes of dynamical behaviour: pathogen extinction, pathogen persistence in a growing population, the pathogen regulating the host population to a stable equilibrium and host–pathogen limit cycles. Conditions are given for the system to be in each of these regions, and the authors conclude that ‘highly pathogenic microparasites producing very large numbers of long-lived infective stages are likely to lead to non-seasonal cyclic changes in the abundance of their invertebrate hosts and in the prevalence of infection’ (pp. 482–483). This model is then discussed in greater detail, and diagrams are presented showing how the periods of limit cycles, when they occur, vary with the model parameters. The model is applied to the dynamics of the larch budmoth, *Zeiraphera diniana*, and its infection with a granulosis virus. The agreement between model and data is said to be encouraging, and the authors conclude that the model is ‘sufficient to account at least for most long-term population cycles in forest insects’ (p. 490). A similar analysis, with a more extensive review of parameters, was presented by Anderson & May in 1980 [27].

Population cycles of forest insects are a favourite example in teaching dynamical systems (e.g. [28]). The reason for the outbreaks is invariably presented as a result of hysteresis generated by the fast timescale of the insect population and the slow timescale of the trees [29]. Pathogens are not usually implicated. Bowers *et al*. [30] extended Model G by replacing exponential growth in the absence of pathogen with logistic population dynamics. Their analysis concluded that host–pathogen interactions by themselves could not generate the observed patterns, although they may contribute to their generation. The argument was taken up again by Berryman [31]. His view was that the observed cycles were due to interactions with insect parasitoids, rather than with a virus or with the forest foliage.

### (e) Dynamics in a fluctuating environment

In their next section, the authors discuss the persistence of microparasites in fluctuating host populations. Here we move away from host–pathogen interactions giving rise to cyclic behaviour, but focus on the characteristics of the pathogen that enable it to survive fluctuations in host population density. The discussion is centred around the mechanisms that may have evolved to enable microparasites to survive when the host population size fluctuates below the critical value for pathogen maintenance, *H*_T_. In the first part of this discussion, host population fluctuations are modelled as seasonal, but the authors then consider cycles generated by interactions between the host and slowly regenerating food supplies. This brings us back to the spruce budworm example. Clearly, the authors favour long-lived infective stages being an effective strategy for pathogen survival. The idea of threshold population size was an important one to emerge from the paper, and is highlighted in their Table 10 in their conclusions. This was not the last word on the subject, and the discussion has continued for more than 20 years [32].

### (f) Biological control and evolutionary trends

Biological control of an insect pest was not a new idea in 1981; substantial modelling effort had been devoted to the problem from the early 1970s, for example by Whitten and collaborators [33]. Prout [34] provides an overview of the literature at the time, where the efforts were focused on genetic control via sterile males; the paper is an elaborate study of the release of sterile males in a density regulated population, using a ‘philosophy’ similar to that of the Anderson & May paper of using simple models and detailed analysis, but focusing on models with discrete generations. This literature is not cited by Anderson & May in 1981, but particularly Prout [34] would have deserved a mention. As far as we are aware, there was relatively little effort before their paper to model biological control using infectious agents.

In the section on biological control in the paper, it is shown that the host population would be driven to extinction if free-living infective stages of the pathogen were introduced at a rate exceeding 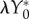, where 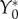 is the equilibrium population size of infected hosts (with no biological control). The authors find it plausible that both these quantities could be estimated, but as we have seen the value of *λ* should be subject to a rescaling. The authors do issue caveats, including a note that no allowance has been made for spatial heterogeneity. While they have movement of the host population in mind, this does raise the question of appropriate scaling and units in all of the models. The authors also note that pathogens exert a selection pressure on their hosts, hence the control measure is aimed at a moving target. This leads into the final section of the paper.

Section 15 on evolutionary trends is perhaps the least convincing part of the paper. It is postulated that the ‘production of transmission stages typically entails some sort of sexual process, where genetic exchange occurs’ (p. 499). This does not sit well with the exposition of microparasite transmission, and no mention of mutation is made. The authors confess that the models in this section are very preliminary, but come to the conclusion that a parasite's optimum strategy is one of intermediate pathogenicity: a concept now widely accepted. Although they contradict themselves in saying that pathogens have a shorter generation time than their hosts, having already determined that to be a counter-productive strategy, they conclude that for invertebrates host and pathogen coevolve. This preliminary analysis paved the way for a much more detailed exposition that appeared the following year [35].

## 3. Impact and present-day developments

The volume and diversity of the literature on infectious disease dynamics, and the extent of its methodology and insights are impossible to sketch here. Instead, we very briefly highlight recent advances related to three dimensions of Anderson & May's paper. The most direct is the topic of infectious diseases of invertebrate hosts. The second is to take the applied view and look at biological control. The third is a view of the integration of ecological and epidemiological questions.

While a substantial part of the study of infectious agents in invertebrate hosts views the hosts as pest species, understanding the role of infectious agents in regulating invertebrates is rapidly becoming a conservation issue. This relates to the potential loss of ecosystem services provided by invertebrates, most notably those species essential as pollinators [36]. The role of pathogens in bee colony collapse and bee decline is an area of intense analysis, including the use of population models [37–40]. It is also an area that has interesting parallels to the situation following Anderson & May's paper, given their new hypothesis and analysis trying to explain the long-term cycles of outbreaks of forest pests such as the larch budmoth. Many different hypotheses and contributing factors have been advanced, of which the role of infectious disease agents is one. A recent point of view from the inherent dynamics of complex systems, when a factor of influence changes slowly leading to population collapse [41], could apply to either area. With regard to recurrent outbreaks of insect pests, similar analyses have recently been presented, for example for the tea tortrix *Adoxophyes honmai* [42].

The dimension of biological control is still relevant today, and the issue has broadened beyond the scope envisaged in the Anderson & May paper. On top of their role as pest species, invertebrates have received growing attention in recent decades for their role as vectors for infectious diseases of animals and humans. In the paper, the idea was to study a species of infectious agent A, which could directly regulate the population of an invertebrate pest species, either preventing or reducing the size of outbreaks of the pest (i.e. host) species. One criterion could be to prevent the reproduction number of the invertebrate species from exceeding one. There is currently a different but related interest in using regulation by an infectious agent A to reduce an invertebrate population to such a level that the reproduction number of another infectious agent B, for which the invertebrate is a vector, is reduced below one. The difference is that in the former case one is only interested in depressing the host population, whereas in the latter case it may be that the infectious agent reduces the competence of individuals of the vector species in transmitting pathogen B. Competence could be reduced, for example, by reducing the lifespan of the invertebrate when infected by pathogen A, thereby reducing the infectious period for transmitting B, or by interfering with replication of pathogen B within a host that is also infected with pathogen A. Attempts to use the bacterial species *Wolbachia pipientis* to control *Aedes aegypti* mosquitoes, the main vector transmitting dengue virus between humans, are important recent examples [43] (for a wealth of information and a brilliant cartoon of the main idea see http://www.eliminatedengue.com/en/program). The models used in the Anderson & May paper would need significant modification to deal with these issues and the subtleties involved. Not least, the human host population for the invertebrate species should be included, as this is involved in gauging the effect of biological control by pathogen A on transmissibility of pathogen B.

Before the 1980s, infectious disease epidemiology had long been focused on understanding the interaction of a single infectious agent in a population of a single host species and has thrived because of this focus. Almost exclusively, and understandably, these species were either humans or farm animals, with much less attention paid to plants and wildlife. During the 1980s, ecological aspects were studied, for example, to understand the dynamics of infections in wildlife and consequences for wildlife conservation; however, a one-on-one interaction prevailed. In recent decades, ecologists have taken a more structured approach to infectious disease agents, studying these agents in multihost settings, and more recently in ecosystems where host species and non-host species of specific infectious agents interact ecologically. Interactions between ecology and epidemiology, particularly in food webs and ecosystems, give rise to many interesting phenomena, and empirically studied systems are abundant [44]. Theoretical studies have concentrated on infectious agents in systems consisting of one predator and one prey species. From the work initiated by May on ecosystems and stability before he (also) became interested in epidemiology, it has emerged that organization and weak interactions in food webs and ecosystems can have decisive effects on stability, even in complex systems [45]. This raises the question whether the incredibly abundant organisms that are infectious agents may, even if they have co-evolved in ecosystems to interactions that are weak with most host species, have important roles in ecosystem structure and stability that have hitherto been unexplored. To study this, one needs a more structured approach to studying infectious agents in ecosystems, a topic that is likely to attract substantial attention from ecologists and mathematical modellers in coming decades [46]. Such studies are also needed if we are to understand the emergence of infectious agents in new hosts (such as humans) from co-evolved ecosystem settings, especially in response to anthropogenic changes in these ecosystems. Some 40 years after May first addressed the stability of multispecies communities, and some 30 years after he and Anderson started to transform infectious disease epidemiology, the two fields can now be merged and studied in a way that would satisfy them both.

The study of infectious disease dynamics has grown into a proper active and important field of research. Although many researchers have contributed to its genesis, the many ways in which Anderson and May contributed have been essential. They were very active in research, with a keen eye for areas that needed attention, frequently sparking interest by a wider community—reflecting the view by May of himself as an ‘R-selected researcher’, quickly exploring new territory before moving on. They interacted with biologists, and later the medical and public health communities. They organized influential meetings (e.g. [47,48]), training a generation of influential epidemiological modellers (and indirectly an ever growing second generation). Of course, more people in the field combined several or all of these qualities, but never this extensively or effectively. And most of that first generation would admit to being influenced and inspired by the five seminal publications of which this 1981 paper was one.

## Author profiles

**Hans Heesterbeek** is professor of Theoretical Epidemiology at Utrecht University. His current research interest is on the dynamics of infectious agents in food webs and ecosystems, as well as on the history of infectious disease epidemiology. He is a co-author of two textbooks on mathematical modelling of infectious diseases and is currently writing a book on the history of the study of epidemics. He is a founder and editor-in-chief (with Neil Ferguson) of the journal *Epidemics*, and editor of *Proceedings of the Royal Society B*. He has known May and Anderson since 1993, when he was a postdoc with Robert May at the department of Zoology of the University of Oxford at the time that Roy Anderson became head of department. In the same year, he met his current co-author Mick Roberts, with whom he has very productively collaborated ever since.

**Mick Roberts** is Professor in Mathematical Biology at Massey University, Auckland, New Zealand. His research interest is to understand the epidemiology of infectious diseases and optimize strategies for their control, using modern methods of mathematical analysis and developing new methods as necessary. He was previously a scientist at the Wallaceville Animal Research Centre near Wellington, where he was the leader of programmes in modelling infectious diseases and parasitology. He has held visiting fellowships at Oxford, Cambridge and Utrecht Universities. Professor Roberts was elected a Fellow of the Institute of Mathematics and its Application (FIMA) in 1992, and a Fellow of the Royal Society of New Zealand (FRSNZ) in 2008. He received the New Zealand Mathematical Society Research Award in 2006. He is the author of over 120 peer-reviewed publications, including more than 90 journal articles.
